# Effect of central corneal curvature on corneal material stiffness parameter acquired by dynamic corneal responses

**DOI:** 10.3389/fbioe.2023.1237834

**Published:** 2023-10-18

**Authors:** Zhe Chu, Qi Ren, Wenjie Su, Wei Cui, Jie Wu

**Affiliations:** ^1^ Eye Institute of Shandong First Medical University, Qingdao Eye Hospital of Shandong First Medical University, Qingdao, China; ^2^ State Key Laboratory Cultivation Base, Shandong Provincial Key Laboratory of Ophthalmology, Qingdao, China; ^3^ School of Ophthalmology, Shandong First Medical University, Qingdao, China

**Keywords:** corneal material stiffness, dynamic corneal response, stress-strain index, corneal curvature, axial length

## Abstract

The stress–strain index (SSI) is a measure of corneal material stiffness, which is obtained using the Corvis ST algorithm based on dynamic corneal response parameters. The reduced SSI corresponds to the longer axial length (AL). In a previous study, we found SSI increases as the corneal curvature flattens, whereas a flatter corneal curvature indicates a longer AL (emmetropia or myopia). Therefore, in this cross-sectional study, we aimed to address these contradictory findings. First, we characterized the features of SSI, curvature radius of the anterior corneal surface (CR), and AL and analyzed their correlation with advanced myopia. Next, we compared the relationship between AL and SSI after adjusting for the effect of CR. We found a significant positive correlation between SSI and CR, which contradicts the developmental law of axial myopia. Furthermore, after accounting for the effect of CR, we observed a stronger correlation between SSI and AL than that in the unadjusted model. In conclusion, CR is an independent influencing factor for SSI in addition to AL, which masked the decrease in SSI caused by prolonged AL in axial myopia.

## 1 Introduction

The biomechanical properties of the cornea are often described using parameters such as material modulus and structural stiffness (Kling and Hafezi, 2017). The Corvis ST system measures these properties by evaluating the dynamic corneal response parameters (DCRs) of the air pulse, where the stiffness is influenced by the elastic modulus of the cornea and the size and shape of the air-pulse-depressurized corneal surface. Unlike linear elastic materials, the stress–strain curve of the cornea is nonlinear, similar to most collagen-based soft tissues. This nonlinearity makes it challenging to define the mechanical properties of the cornea using Young’s elastic modulus test data alone. The stress–strain index (SSI) is an algorithm predicted by DCRs under air pressure to reflect the entire stress–strain behavior of the corneal material, independent of intraocular pressure and corneal geometry (Eliasy et al., 2019). Previous studies have shown a correlation between reduced corneal stiffness and lower SSI values in myopic eyes (Liu et al., 2021). Myopia is typically associated with elongated eyeballs, but it can also be caused by a highly curved cornea and/or a lens with increased optical power. Refractive myopia is generally considered unrelated to ocular wall stiffness, whereas axial myopia is influenced by it (Flitcroft et al., 2019). Therefore, in myopic eyes, a higher proportion of axial factors leads to decreased ocular wall stiffness and lower SSI values.

However, our previous study revealed a stronger correlation between SSI and spherical equivalent refractive error (SER) or axial length-to-corneal radius ratio (AL/CR) than between SSI and axial length (AL) alone (Chu et al., 2022; Ren et al., 2023). This suggests that the component of refractive myopia also contributes to reduced SSI. To account for this, we adjusted for the refractive myopia component compensated by CR and analyzed the relationship between SSI and axial growth using the average AL during emmetropization, which is determined with ocular refractive power as a reference point. We observed narrower confidence intervals and steeper slopes than those of SSI and AL. Additionally, SSI was found to be proportional to emmetropic AL determined by ocular refractive power, indicating that SSI increases as CR increases (with decreased corneal refractive power and reduced refractive myopia proportion) (Ren et al., 2023). This suggests that factors associated with refractive myopia gradually mask the effect of AL on SSI as myopia progresses.

Therefore, in this cross-sectional study, our aim was to confirm whether the relationship between SSI and CR depends on the dioptric properties of CR, thereby establishing an indirect relationship between SSI and CR. We first characterized the features of SSI, CR, and AL and analyzed their correlation with advanced myopia. Subsequently, we compared the relationship between AL and SSI after adjusting for the effect of CR.

## 2 Methods

This cross-sectional comparative study was conducted at the Eye Institute of Shandong First Medical University in Qingdao, China. The study followed the principles outlined in the Declaration of Helsinki and was approved by the Ethics Committee of the Qingdao Eye Hospital of Shandong First Medical University.

Participants included healthy individuals and patients scheduled for refractive surgery at the Qingdao Eye Hospital of Shandong First Medical University between July 2021 and April 2022. To account for the effect of axial myopia on SSI, participants were grouped based on their AL. The study aimed to investigate the relationship between corneal curvature and corneal biomechanics in individuals with myopia; therefore, we excluded participants who met any of the following criteria: 1) astigmatism of 3 diopters (3D) or higher, 2) use of contact lenses, 3) history or suspicion of corneal diseases such as keratoconus, and 4) history of eye surgery. Only data from the right eye of each individual were analyzed due to the high correlation of corneal parameters between the right and left eyes.

To be eligible, participants’ medical records needed to include a complete medical history and results of ophthalmic examinations conducted on the same day, including comprehensive optometry results after mydriasis. The mean anterior corneal curvature radius within a 3-mm diameter range of the corneal apex (CR) and AL were measured using the OA 2000 device (Tomey, Japan). The corneal biomechanical parameter, SSI, was determined using the Corvis ST device (Oculus, Wetzlar, Germany). Only measurements with “OK” quality specifications were included in the analysis.

Statistical analysis and data visualization were performed using R statistical software (version 4.2.2). The significance level was set at *p* < 0.05. Continuous variables were summarized as mean (standard deviation), while categorical variables were presented as sample size (percentage). Pearson’s correlation tests were conducted to examine the relationships between SSI, AL, and CR, and a matrix of plots was created to analyze the correlations between these three variables. Linear regression models were used to examine the associations between the variables: Model 1 treated SSI as the dependent variable and AL as the independent variable, Model 2 included an additional adjustment for CR, and Model 3 further adjusted for the AL×CR interaction term. The Akaike Information Criterion (AIC) (Akaike, 1974) and model likelihood ratio tests (King, 1989) were performed to compare the model performance of these 3 models. The model with the lowest AIC values was chosen as the best-fitting model with the best tradeoff between the goodness of fit and complexity of the model. The AIC can be calculated based on the following equation: AIC = 2k—2log-likelihood, where k represents the number of model parameters and log-likelihood represents the log-likelihood of the model. The model likelihood ratio tests were conducted to assess the statistical significance of the differences in model fit. Finally, a subgroup analysis was conducted by categorizing AL into two groups based on a cutoff score (AL < 26 mm vs. AL ≥ 26 mm). In each group, three linear regression models were used, with and without adjusting for CR, using AL as the predictor variable and SSI as the dependent variable. All models were adjusted for age and sex.

## 3 Results

### 3.1 Demographic information and clinical variables on study participants

Table 1 displays the demographic information and clinical variables of our study subjects. A total of 267 participants were included in the present study. As shown in Table 1, continuous variables are summarized as mean (standard deviation), and categorical variables are summarized as sample size (percentage).

**TABLE 1:** Characteristics of our study subjects.

Characteristic	N = 267^a^
Gender	
Female	145 (54%)
Male	122 (46%)
Age, year	22 (8)
SSI	0.82 (0.15)
CR, mm	7.78 (0.24)
AL, mm	26.01 (1.48)
AL (categorical)	
<26 mm	135 (51%)
≥26 mm	132 (49%)

Abbreviations: SSI, stress-strain index; CR, curvature radius of the anterior corneal surface; AL, axial length.

^a^ n (%); Mean (SD).

### 3.2 Correlations between SSI, AL, and CR

**FIGURE 1:**
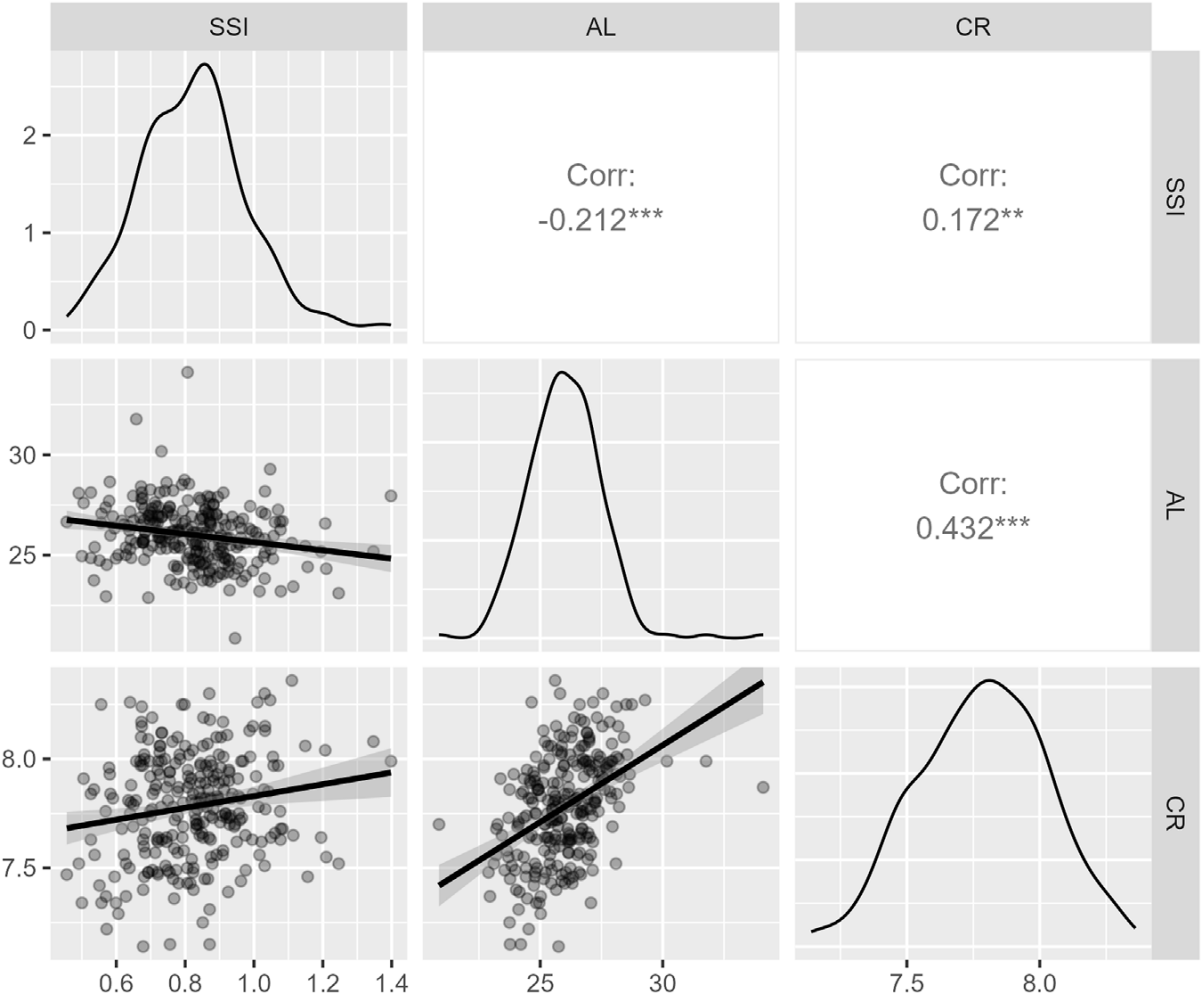
A matrix of plots showing the correlations between SSI, AL, and CR. The units of AL and CR are millimeters. Abbreviations: SSI: stress–strain index; CR, curvature radius of the anterior corneal surface; AL: axial length.

To examine the relationships between SSI, AL, and CR, Pearson’s correlation tests were performed. As shown in Figure 1, the results suggested a negative correlation between SSI and AL (*r* = −0.212, *p* < 0.001), a positive correlation between SSI and CR (*r* = 0.172, *p* = 0.005), and a positive correlation between AL and CR (*r* = 0.432, *p* < 0.001).

### 3.3 Results of linear regression models

As stated in the section on Statistical analyses, three linear regression models were performed to study the associations between SSI, AL, and CR. The results of the three regression models were summarized in Table 2. For Model 1, we found that AL was negatively associated with SSI (*β* = −0.02, *95% CI* = −0.04 to −0.01, *p* < 0.001) after adjusting for age and gender. For model 2, we found that AL was still negatively associated with SSI (*β* = −0.04, *95% CI* = −0.06 to −0.03, *p* < 0.001) after additionally adjusting for CR, while the slope (*β*) changed from −0.02 (model 1) to −0.04 (model 2). In addition, we further performed a linear regression model with an interaction term (AL×CR) to study whether the relationship between AL and SSI was dependent on CR. In Model 3, CR was treated as a continuous variable, while CR was categorized into three groups based on its mean and standard deviation to facilitate the interpretation of the interaction term (Supplementary Figure S1). As shown in Table 2, the interaction term was not significant (*β* = −0.01, *95% CI* = −0.06 to 0.04, *p* > 0.05), indicating that the relationship between AL and SSI was not dependent on CR. The AICs of Model 1, Model 2, and Model 3 were −244.8, −271.4, and −269.5, respectively. The model likelihood ratio tests also suggested that Model 2 was the best-fitting model (Model 2 vs Model 1: F statistics = 29.5, *p* < 0.001; Model 3 vs Model 2: F statistics = 0.1, *p* = 0.74).

**TABLE 2:** Results of linear regression models.

	Model 1	Model 2	Model 3
(Intercept)	1.41 ***	0.05	−1.75
	[1.08, 1.74]	[-0.54, 0.63]	[-12.55, 9.05]
Age, year	0.00	0.00	0.00
	[-0.00, 0.00]	[-0.00, 0.00]	[-0.00, 0.00]
Male gender	0.01	−0.01	−0.01
	[-0.03, 0.04]	[-0.05, 0.02]	[-0.05, 0.02]
AL, mm	−0.02 ***	−0.04 ***	0.03
	[-0.04, −0.01]	[-0.06, −0.03]	[-0.39, 0.44]
CR, mm		0.23 ***	0.46
		[0.15, 0.32]	[-0.92, 1.85]
AL×CR			−0.01
			[-0.06, 0.04]
N	267	267	267
AIC	−244.8	−271.4	−269.5

Notes: N represents the sample size in each model. The 95% confidence intervals were used to describe the uncertainties of coefficients. All coefficients were reported using unstandardized coefficients. ***p < 0.001; **p < 0.01; *p < 0.05.

Abbreviations: SSI, stress–strain index; AL, axial length; CR, curvature radius of the anterior corneal surface; AIC, akaike information criterion.

Finally, additional four linear regression models with SSI as the dependent variable were performed separately for individuals with AL < 26 and AL ≥ 26. The model results are shown in Table 3. Among participants with AL < 26, the unadjusted model (i.e., the model did not include CR) showed that AL was not associated with SSI (*β* = −0.02, *95% CI* = −0.06 to 0.01, *p* = 0.13), while the adjusted model (i.e., the model included CR) showed that AL was negatively associated with SSI (*β* = −0.05, *95% CI* = −0.09 to −0.02, *p* < 0.01). However, among participants with AL ≥26, we did not observe a significant association between AL and SSI neither in the unadjusted model (*β* = −0.02, *95% CI* = −0.04 to 0.01, *p* = 0.25) or in the adjusted model (*β* = −0.03, *95% CI* = −0.05 to 0.00, *p* = 0.0591).

**TABLE 3:** Results of a subgroup analysis.

	Participants with AL < 26		Participants with AL ≥ 26	
	Unadjusted	Adjusted	Unadjusted	Adjusted
(Intercept)	1.49 ***	−0.04	1.12 **	0.12
	[0.71, 2.28]	[-1.00, 0.92]	[0.45, 1.80]	[-0.90, 1.13]
Age, year	−0.00	0.00	0.00 *	0.00 *
	[-0.01, 0.00]	[-0.00, 0.00]	[0.00, 0.01]	[0.00, 0.01]
Male gender	0.04	0.01	−0.03	−0.05
	[-0.02, 0.09]	[-0.04, 0.06]	[-0.08, 0.02]	[-0.10, 0.01]
AL, mm	−0.02	−0.05 **	−0.02	−0.03
	[-0.06, 0.01]	[-0.09, −0.02]	[-0.04, 0.01]	[-0.05, 0.00]
CR, mm		0.28 ***		0.16 *
		[0.17, 0.40]		[0.04, 0.29]
N	135	135	132	132

Notes: N represents the sample size in each model. The 95% confidence intervals were used to describe the uncertainties of coefficients. All coefficients were reported using unstandardized coefficients. The term “Unadjusted” indicates that the models did not include CR, while the term “Adjusted” indicates that the models included CR. ***p < 0.001; **p < 0.01; *p < 0.05.

Abbreviations: SSI, stress–strain index; AL, axial length; CR, curvature radius of the anterior corneal surface.

## 4 Discussion

This study aimed to investigate the correlation between central corneal curvature and corneal biomechanical behavior parameters acquired using DCRs from a dioptric perspective, to address conflicting conclusions reported in previous studies regarding the relationship between SSI and AL in myopic eyes. For this purpose, linear regression models were established to examine the associations between the variables. The comparison of model performance using AIC and model likelihood ratio tests provided valuable insights into the performance of the three models (Table 2). Based on the AIC, Model 2 exhibited the lowest value, indicating the best fit to the data among the three models. Furthermore, the model likelihood ratio tests also supported the superiority of Model 2 over the other models. Taken together, our findings suggested that Model 2 (including AL and CR as independent variables) captures the data patterns more effectively than Models 1 and 3. Finally, the study found a positive correlation between SSI and CR, contrary to the pattern observed with deepening axial myopia. After adjusting for the effect of CR, the correlation between SSI and AL became more significant, particularly when AL was <26 mm.

DCRs obtained from Corvis ST are influenced by internal structures and scleral stiffness (Kling and Marcos, 2013). SSI has been reported to be associated with reduced biomechanical strength of the cornea related to myopia, and it has been speculated that the correlation between SSI and AL is due to the influence of scleral biomechanics. However, interpreting the relationship between CR and SSI is more complex. First, in axial myopia, the biomechanical strength of the ocular wall including the cornea decreases (Sedaghat et al., 2020), whereas the anterior corneal surface curvature flattens rapidly and eventually stabilizes (Gonzalez Blanco et al., 2008; Jin et al., 2022). In this study, both CR and SSI changed in a unidirectional manner with increasing axial myopia, with CR increasing and SSI decreasing, consistent with theoretical expectations. Second, the absence of a causal relationship between the degree of refractive myopia and the biomechanical strength of the cornea suggests that the correlation between CR and SSI may be indirect, based on the deepening of axial myopia, i.e., a higher CR would correspond to a lower SSI. However, we found the opposite result, indicating that the correlation between CR and SSI was not based on the indirect correlation observed with increasing AL. Third, the linear regression models used in the study demonstrated that the relationship between AL and SSI was not dependent on CR, suggesting that CR is an independent influencing factor for SSI.

The SSI algorithm was developed by using the least squares method based on numerical modeling input and output parameters CCT, bIOP, and stiffness parameter at highest concavity (SP-HC), while corneal curvature was fixed at 7.8 mm (Eliasy et al., 2019). Therefore, after correcting for a wide range of CCT and bIOP values by the least squares method, SSI may still be affected by CR through SP-HC. As a DCR-based stiffness parameter, SP-HC is defined as the final load at corneal pressure divided by the difference between the corneal deflection and the maximum depression value at that time, reflecting the structural properties of the cornea. Differences in corneal curvature can lead to an uneven distribution of air-puff-related stress, resulting in unaccounted strain and variations in SP-HC. While corneal geometry may influence the acquisition of SSI to some extent, the SSI itself should not be correlated with corneal geometry since it is a material index (Eliasy et al., 2019). Considering that the cornea and anterior sclera of normal eyes reach adult levels by the age of 2 and the posterior sclera does not reach adult levels until the age of 13 (Fledelius and Christensen, 1996; Touzeau et al., 2014), we speculate that the following possibility is more likely: At the end of emmetropization, a flatter cornea may correspond to a higher material stiffness. Following emmetropization, as myopia develops, the ocular wall continues to expand, leading to AL growth in the posterior pole of the sclera and CR increasing. In other words, CR reflects the material stiffness of the anterior structure of the eyeball, whereas AL reflects the posterior pole. Our previous study supported the latter (Ren et al., 2023), but the former still requires confirmation through further research.

In addition, the phenomenon of the better correlation of SSI with SER, AL/CR, and astigmatism of the anterior corneal surface, rather than AL (Liu et al., 2021; Chu et al., 2022; Liu et al., 2022) can be explained to some extent, which was due to a deeper degree of SER with steeper corneal curvature. After adjusting for the effect of CR, the relationship between SSI and AL became more significant than that in the unadjusted model. This suggests that flattened corneal curvature masks the decrease in SSI caused by prolonged AL to some extent. The lower significance observed at AL ≥ 26 mm may be attributed to the confounding effect of posterior scleral staphyloma.

From the perspective of dioptric optics, this study analyzed the relationship between SSI and CR and found it to be contrary to the characteristic changes associated with increasing axial myopia. These results suggest that CR is an independent influencing factor for SSI, in addition to AL, indicating that the acquisition of SSI may be influenced, to some extent, by corneal geometry or that corneal flattening may be related to an increase in the material stiffness of the corneal stroma. Furthermore, we confirmed that the effect of CR did not alter the association between AL and the corneal material stiffness parameter obtained through DCRs. Our findings contribute to understanding the influence of corneal curvature on the development of algorithms for the corneal biomechanical behavior parameter acquired by DCRs. However, it is important to note that the average curvature radius of the anterior corneal surface within 3 mm of the apex, as an essential optometry parameter, cannot fully reflect the characteristics of the noncentral corneal region, especially when it is smaller than the applanation length of the air pulse. Therefore, this finding does not represent the effect of global corneal curvature on SSI acquisition. Given the nature of cross-sectional studies, we cannot exclude the possibility that corneal flattening may be related to the increased material stiffness of the corneal stroma, which could lead to the observed positive correlation between CR and SSI. Future studies should focus on examining the relationship between SSI and the best fitting surface of the anterior and posterior corneal surfaces while controlling for myopia-related variables.

## 5 Conclusion

CR is an independent influencing factor for SSI in addition to AL. CR masks the decrease in SSI caused by prolonged AL in axial myopia.

## Data Availability

The original contributions presented in the study are included in the article/Supplementary Material, further inquiries can be directed to the corresponding author.
