# Supplementary material for: Effect of central corneal curvature on corneal material stiffness parameter acquired by dynamic corneal responses
Source: Front Bioeng Biotechnol. 2023 Oct 18;11:1237834. doi: 10.3389/fbioe.2023.1237834 (PMC10619688; doi:10.3389/fbioe.2023.1237834)
Supplement: Supplementary file 1 [file DataSheet1.docx]

Supplementary Material

## Supplementary Figures


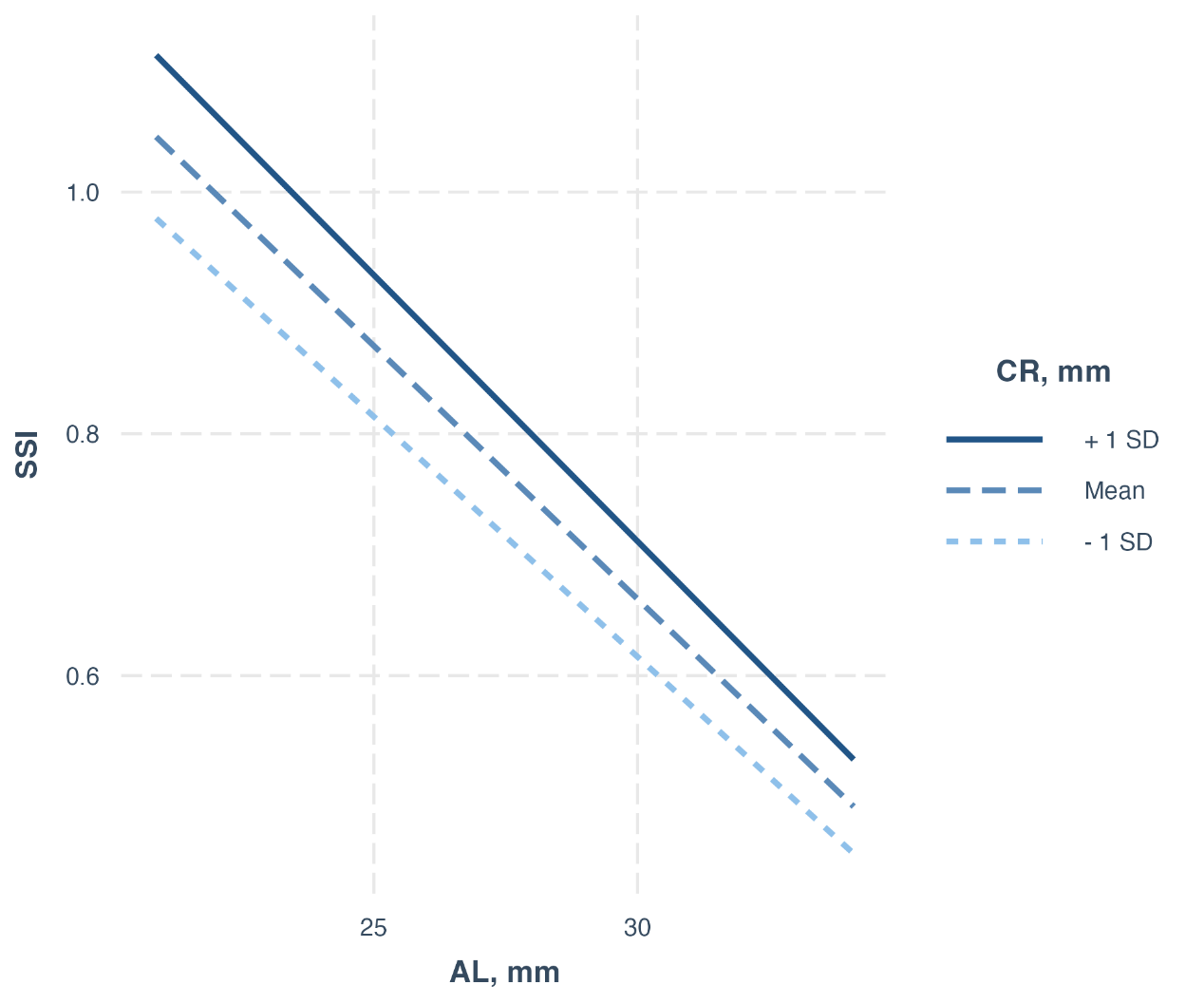


**Supplementary Figure 1.** **Relationships between AL, SSI, and CR**. SD represents standard deviation. Abbreviations: SSI, Stress-Strain index; AL, Axial length; CR: Corneal curvature.
